# Total Bolus Extraction Method Improves Arterial Image Quality in Dynamic CTAs Derived from Whole-Brain CTP Data

**DOI:** 10.1155/2014/603173

**Published:** 2014-07-16

**Authors:** Elyas Ghariq, Adriënne M. Mendrik, Peter W. A. Willems, Raoul M. S. Joemai, Eidrees Ghariq, Evert-jan Vonken, Matthias J. P. van Osch, Marianne A. A. van Walderveen

**Affiliations:** ^1^Department of Radiology, Leiden University Medical Center, Albinusdreef 2, 2333 ZA Leiden, The Netherlands; ^2^Image Sciences Institute, University Medical Center Utrecht, Heidelberglaan 100, 3584 CX Utrecht, The Netherlands; ^3^Department of Radiology, C.J. Gorter Center for High Field MRI, Leiden University Medical Center, Albinusdreef 2, 2333 ZA Leiden, The Netherlands; ^4^Department of Radiology, University Medical Center Utrecht, Heidelberglaan 100, 3584 CX Utrecht, The Netherlands

## Abstract

*Background and Purposes*. The 320-detector row CT scanner enables visualization of whole-brain hemodynamic information (dynamic CT angiography (CTA) derived from CT perfusion scans). However, arterial image quality in dynamic CTA (dCTA) is inferior to arterial image quality in standard CTA. This study evaluates whether the arterial image quality can be improved by using a total bolus extraction (ToBE) method. *Materials and Methods*. DCTAs of 15 patients, who presented with signs of acute cerebral ischemia, were derived from 320-slice CT perfusion scans using both the standard subtraction method and the proposed ToBE method. Two neurointerventionalists blinded to the scan type scored the arterial image quality on a 5-point scale in the 4D dCTAs in consensus. Arteries were divided into four categories: (I) large extradural, (II) intradural (large, medium, and small), (III) communicating arteries, and (IV) cerebellar and ophthalmic arteries. *Results*. Quality of extradural and intradural arteries was significantly higher in the ToBE dCTAs than in the standard dCTAs (extradural *P* = 0.001, large intradural *P* < 0.001, medium intradural *P* < 0.001, and small intradural *P* < 0.001). *Conclusion*. The 4D dCTAs derived with the total bolus extraction (ToBE) method provide hemodynamic information combined with improved arterial image quality as compared to standard 4D dCTAs.

## 1. Introduction

Cerebral computed tomography perfusion (CTP) scans are acquired in patients with acute stroke [1] or subarachnoid hemorrhage [2]. Although there is some debate [2–5] about the prognostic value of CT perfusion, many studies [1, 2, 6–9] have shown that it provides valuable information about the cerebral hemodynamics, especially with the introduction of 320-slice CT scanners (16 cm coverage) that enable the acquisition of whole-brain CTP scans and provide an option to derive 4D dynamic CT angiography (dCTA) images from the CTP scans [9–12]. These 4D dCTA images show great potential for the assessment of collaterals [13], the measurement of cerebral circulation times [14], and arteriovenous shunting lesion assessment [15]. However, it has been shown that the 4D dCTAs have inferior quality compared to standard 3D CTA scans [16]. Therefore, cerebral arteries, and in particular arteries with a small diameter, are more difficult to assess. Several methods have been proposed to derive vascular information from CTP scans [17–19] of which some are with a quality comparable to standard 3D CTA [19, 20]. However, these methods do not provide full 4D dynamic CTA images but instead provide a 3D image showing the vasculature. Therefore, even though the arterial image quality is good, the hemodynamic information available in the 4D dCTA images is lost. In this paper, we propose a total bolus extraction (ToBE) method to derive 4D dCTAs from CTP scans in which the hemodynamic information is preserved. We performed an observer study in which two neurointerventionalists scored the image quality of the intracranial arteries in 30 anonymized and randomized dCTAs (15 standard and 15 ToBE dCTAs). The observers were blind to the type of dCTA that was presented to them. Our hypothesis is that the ToBE method will improve the arterial image quality in the 4D dCTAs derived from the 4D CTP scans compared to the standard method to derive 4D dCTAs.

## 2. Materials and Methods

### 2.1. Patients

Fifteen patients (4 males, 11 females; average age of 70.2 years, range 53–88 years) were included from a large prospective multicenter observational cohort study. This cohort study evaluates the predictive value of CTP and CTA on the clinical and radiological outcome measures of patients presenting with symptoms of acute ischemic stroke. On admission, all patients underwent an unenhanced CT and a CTP study of the brain using Toshiba Aquilion ONE 320-slice CT scanner (Toshiba Medical Systems, Otawara, Japan). Inclusion criteria were age >18 years, onset of stroke symptoms <9 hours, National Institutes of Health Stroke Scale (NIHSS) ≥2, and informed consent from patient or family. Exclusion criteria were known renal failure and contrast allergy.

### 2.2. CTP Scan Acquisition Protocol

CTP studies were acquired during injection of 50 mL of contrast agent (IOMERON 400, BRACCO Imaging Europe) with a flow rate of 5 mL/s followed by 40 mL saline solution with a flow rate of 4 mL/s using an antecubital placed intravenous line. A total of 24 volume scans were acquired (slice thickness: 0.5 mm, slice interval: 0.5 mm) with a total scan time of around three and a half minutes. The CTP acquisition procedure consisted of a mask volume (5 seconds after start of contrast agent administration), followed by a 10-second delay, 4 early arterial volumes (80 mA, 80 kV), 6 full dose arterial volumes (300 mA, 80 kV), and 3 late arterial volumes with an interscan delay (ISD) of 2 seconds (160 mA, 80 kV). Next, 4 venous volumes (ISD of 5 seconds) and 6 delayed volumes starting at 90 seconds (ISD of 30 seconds) were obtained (130 mA, 80 kV).

### 2.3. Postprocessing

#### 2.3.1. Standard 4D Dynamic CTA

The standard 4D dCTAs were derived from the 320-slice CTPs on a postprocessing workstation (Vitrea fX, Vital Images, Minnetonka, USA). This software subtracts the first unenhanced volume of the CTP study from the subsequent contrast enhanced volumes to ensure that only vessels remain visible. From the resulting dCTA, the volume with the highest arterial contrast (arterial phase) was selected for quality analysis of the arteries.

#### 2.3.2. ToBE 4D Dynamic CTA

ToBE dCTAs were derived from the 320-slice CTPs by employing the total bolus extraction method developed in the UMC Utrecht, The Netherlands. This method is based on a method proposed by Mendrik et al. [18, 20] that derives 3D angiograms from CTP data. These 3D angiograms (vessel enhanced volumes) were derived from the CTP data by quantifying the total change in Hounsfield units (HU) over all temporal volumes (total bolus), using the absolute area under the first temporal derivative curve as described in [18]. For this study, dynamic information extracted from the original CTP data was added to the 3D angiograms, to create the ToBE dCTAs, as follows: a Gaussian temporal filter (*σ* = 1 sec) was applied to the original CTP data to reduce noise. Subsequently, the time-intensity curves in the filtered CTP data were normalized to values between zero and one, by detecting the baseline and maximum, and multiplied by the 3D angiogram. An automatically generated skull mask was used to mask possible registration artifacts. From the resulting ToBE dCTA, the volume with the highest arterial contrast (arterial phase) was chosen for quality analysis of the arteries.

### 2.4. Quality Analysis

A total of 30 (15 standard dCTAs, 15 ToBE dCTAs) maximum intensity projections (MIPs) were created, anonymized, and randomized. Quality analysis of the arteries on these MIP-CTAs was done by two neurointerventionalists in consensus. Arteries were divided into four subcategories: (I) large extradural arteries (internal carotid arteries (ICAs) and vertebral arteries (VAs)); (II) intradural arteries: large intradural arteries (basilar artery (BA), first segments of anterior cerebral artery (A1), middle cerebral artery (M1), and posterior cerebral artery (P1)), medium sized intradural arteries (A2, P2, and P3), and small sized intradural arteries (A3, A4, M2, M3, M4, and P4); (III) communicating arteries (anterior communicating (Acom) and posterior communicating (Pcom) arteries); and (IV) cerebellar (superior cerebellar artery (SCA) and anterior inferior cerebellar artery (AICA)) and ophthalmic arteries. A 5-point analogue scoring system (5 = good quality, 1 = poor quality) was used for evaluation of image quality of the following arteries: VA, ICA, BA, SCA, AICA, ophthalmic artery, A1, Acom, A2, M1, P1, P2, Pcom, and P3. The following image quality characteristics were evaluated: arterial contrast, arterial contour sharpness, and regularity and impression of contrast to background noise ratio. Since segments of small intradural arteries (A3, A4, M3, M4, and P4) contain several peripheral branches, it is nearly impossible to provide an overall quality score for each individual vessel segment. Therefore the observers had to indicate whether 3 or more peripheral branches in each vessel segment were visible or not, with respect to the aforementioned image quality characteristics. This binary (>3 branches visible yes/no) scoring system is also used for the M2 segment of MCA, since this segment also divides into several branches directly after its division from the M1 segment; for this reason, the M2 segment is defined and scored as a small intradural artery in this study (although in terms of vessel diameter the M2 segment would be categorized in the group of medium sized intradural arteries). The cerebellar and ophthalmic arteries were scored as a separate group on the 5-point scale and no distinction between small and medium sized arteries was made. The reason for this is that the ramifications of the cerebellar and ophthalmic arteries are less well defined and balanced as compared to the small sized intradural arteries, and the arteries lack proximal divisions (e.g., the ophthalmic artery branches only when it crosses over the optic nerve). The communicating arteries were scored separately on the 5-point scale as well, because they can vary considerably in presence and size, due to, for example, aplasia, hypoplasia, or fetal continuation of the posterior cerebral artery, which complicates the classification of these arteries as medium or small.

### 2.5. Statistical Analysis

To compare the arterial image quality in the standard dCTAs and the ToBE dCTAs, paired nonparametric statistical tests were used. For the small intracerebral arteries, the McNemar test was used and the Wilcoxon Signed Rank test was used for the remaining arteries. The statistical analysis was performed using the SPSS 18 software package (IBM SPSS Inc., Chicago, IL, USA). A significance level of *α* = 0.002 was chosen after Bonferroni correction for multiple comparisons.

## 3. Results

**Figure 1:**
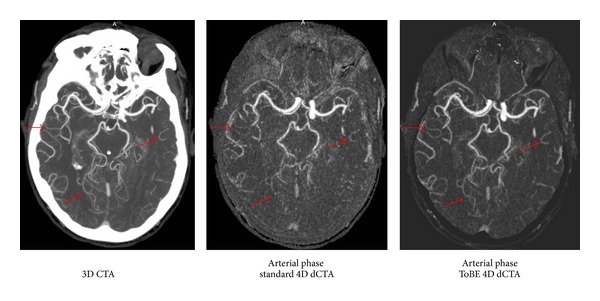
Illustration (maximum intensity projections over a 15 mm axial slab) of the image quality of the arteries in the dynamic 4D CTAs compared to the 3D CTA. The arrows indicate the arteries that show improved arterial image quality in the ToBE 4D dCTA compared to the standard 4D dCTA. The 3D CTA can be used as a reference.

The results of the observer study are presented in Table 1. Some arteries were excluded from the statistical evaluation due to the following findings: ICA occlusion (2), BA occlusion (1), A1 occlusion (1), AICA occlusion (3), ophthalmic artery occlusion (3), and Pcom aplasia (4). The final clinical diagnoses included transient ischemic attack (4) and cerebral ischemia (7). In 4 patients, the definite diagnosis remained uncertain. The results show that the arterial image quality in the ToBE dCTAs was scored significantly higher than arterial image quality in the standard dCTAs for the large extradural and the large and medium intradural arteries. The number of visible artery branches of the small intradural arteries also improved significantly in the ToBE dCTAs compared to the standard dCTAs. Although the mean scores for the communicating arteries and cerebellar and ophthalmic arteries were slightly higher in the ToBE dCTAs compared to the standard dCTAs, this difference was not significant.  Figure 1 illustrates the quality of the evaluated arterial phase in the 4D dCTAs compared to the 3D CT angiography (CTA) scan.

**Figure 2:**
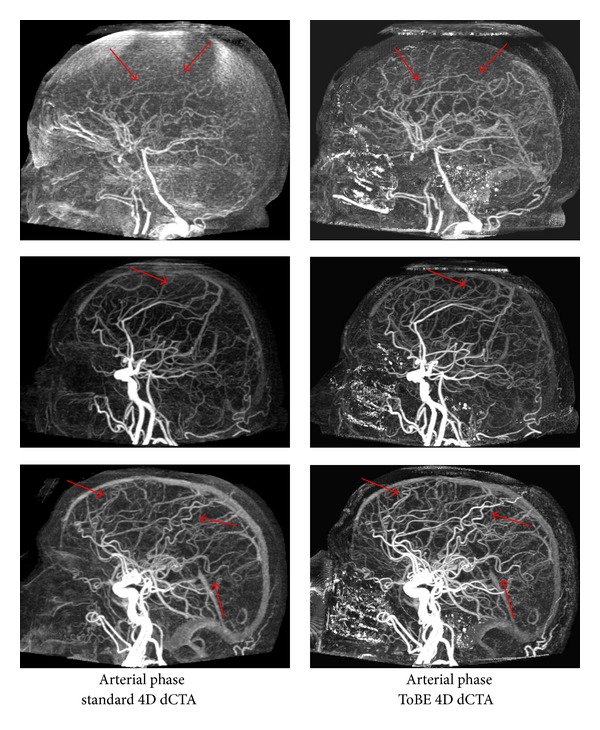
Illustration of the arterial phase in the dynamic CTAs derived from the CTP data. The arterial phase (whole-brain maximum intensity projection) is shown of both the standard and ToBE dCTAs of three of the evaluated 15 subjects. The arrows indicate locations where the ToBE dCTAs show improved visualization of the arteries.

**Figure 3:**
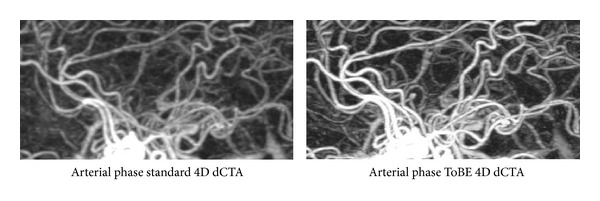
Zoomed in subimages of the arterial phase in one of the evaluated subjects in both the standard 4D dynamic CTA (dCTA) and the proposed ToBE 4D dCTA.

Figure 2 shows three examples of the improved arterial image quality in the arterial phase of the ToBE dCTA as compared to the standard dCTA.  Figure 3 shows a zoomed in subimage to illustrate the enhancement of the small arteries.

## 4. Discussion

The 320-slice CT scanner has introduced whole head coverage in a single rotation enabling acquisition of whole-brain CT perfusion (CTP) scans at a high temporal resolution. Consequently, whole-brain 4D dynamic CTAs (dCTAs) can be derived from these CTP scans that provide information on the cerebral hemodynamics. Unfortunately, the arterial image quality of these 4D dCTAs has been shown to be inferior to 3D CTA [14]. In this paper, we proposed the total bolus extraction (ToBE) method as an alternative to the standard subtraction-based method to derive 4D dCTAs from CTP scans. The standard method subtracts the first unenhanced volume of the CTP scan from each of the subsequent volumes to derive the dCTA image, which increases noise and could decrease the arterial image quality. The ToBE method uses all volumes available in the CTP scan to retrieve the total amount of available contrast for each vessel (total bolus), after which the hemodynamic information from the CTP scan is added by showing the corresponding percentage of the total bolus at each point in time. Our hypothesis was that the ToBE method would improve the arterial image quality in 4D dCTAs compared to the standard method. Our observer study confirmed this hypothesis for the large extradural arteries and the intradural arteries. In these arteries, the image quality improved significantly. The quality of the communicating arteries and cerebellar and ophthalmic arteries showed slight improvements as well, but these were not significant. The improved arterial image quality in the ToBE dCTAs was especially prominent for the small intracranial arteries. Therefore, ToBE dCTAs could have the potential to improve detection of small-artery pathologies, for example, vasculitis, small distal aneurysms, and small shunting lesions, as compared to standard dCTAs, since Siebert et al. [16] reported that the evaluation of the medium and small intracranial arteries in standard dCTAs is limited, due to the inferior quality compared to 3D CTA.

Although the results of our study are promising, there are some limitations. First, in this study we did not compare the arterial image quality of the ToBE 4D dCTA with the arterial image quality of standard 3D CTA. We cannot therefore be certain that the ToBE 4D CTA would have the potential to replace the 3D CTA in the diagnostic work-up of ischemic stroke patients and thus may limit radiation exposure to patients. This comparison will be the subject of future studies. Second, we did not include patients with pathology of small arteries; we cannot therefore be sure that improvement of image quality of small cerebral arteries by applying the ToBE method is sufficient for detecting pathologies in these arteries. The arterial image quality improved, but further research in patients with pathologies of small arteries should assess whether the small arteries are indeed diagnostic. Third, a relatively small number of patients were used to evaluate the ToBE method. Finally, the skull mask that was used for the ToBE method introduced some artifacts in the arteries near the cranial base, that were not present in the standard dCTAs, since this method does not use skull masking. A solution to this problem would be to use the ToBE method without bone masking, since bone is automatically suppressed [18, 20]. However, in this study we used the bone mask to suppress high intensities that could result from small misalignments of the sequential CTP volumes and might reduce depiction of arteries. Improving the registration method could resolve this limitation, which is a subject for further research.

Despite these limitations, the ToBE dCTAs showed improved image quality of especially the small intracranial arteries as compared to the standard dCTAs. The clinical significance of the improved image quality of the small intracranial arteries needs to be validated in patients with small vessel pathology, for example, compared to digital subtraction angiography as a reference standard.

## 5. Conclusion

In conclusion, this study showed that 4D dynamic CTAs derived from 320-slice CTP scans using the total bolus extraction (ToBE) method show improved image quality of the large extradural and all intradural arteries as compared to standard 4D dynamic CTAs derived using the subtraction method. Arterial image quality improvements were especially prominent in the small intradural arteries. The 4D ToBE dynamic CTAs derived from the CTP scans combine valuable hemodynamic information with improved arterial image quality.

## Figures and Tables

**Table 1:** Results of the observer study on arterial image quality in standard dynamic CTAs (dCTAs) and the proposed ToBE dynamic CTAs derived from 15 4D CT perfusion scans. The scores are presented as mean (standard deviation) over all 15 images and were scored on a 5-point scale (5 = good quality, 1 = poor quality) for all arteries, except the small intradural arteries. The small intradural arteries are presented as the number of scans with >3 visible small artery branches/total number of small arteries assessed (ratio in percentage; standard error).

Category	Standard dCTAs	ToBE dCTAs	Test results
Large extradural arteries	4.5 (0.7)	4.9 (0.4)	*P* = 0.001*
Intradural arteries			
Large	4.4 (1.0)	4.7 (0.7)	*P* < 0.001*
Medium	3.7 (1.1)	4.4 (0.8)	*P* < 0.001*
Small	104/180 (57.8%; 3.7%)	159/180 (88.3%; 2.4%)	*P* < 0.001**
Cerebellar and ophthalmic arteries	2.5 (1.6)	2.9 (1.7)	*P* = 0.007
Communicating arteries	2.7 (1.8)	2.9 (1.9)	*P* = 0.10

*Significant, Wilcoxon Signed Rank statistical test.

**Significant, McNemar statistical test.

## Abbreviations

ToBE: Total bolus extraction method
Acom: Anterior communicating artery
Pcom: Posterior communicating arteries
AICA: Anterior inferior cerebellar artery
SCA: Superior cerebellar artery
dCTA: Dynamic 4D whole-brain CT angiograms derived from CTP scans.

## References

[1] Zhu G, Michel P, Aghaebrahim A (2013). Computed tomography workup of patients suspected of acute ischemic stroke: perfusion computed tomography adds value compared with clinical evaluation, noncontrast computed tomography, and computed tomography angiogram in terms of predicting outcome. *Stroke*.

[2] Cremers CHP, van der Schaaf IC, Wensink E (2013). CT perfusion and delayed cerebral ischemia in aneurysmal subarachnoid hemorrhage: a systematic review and meta-analysis. *Journal of Cerebral Blood Flow & Metabolism*.

[3] Goyal M, Menon BK, Derdeyn CP (2013). Perfusion imaging in acute ischemic stroke: let us improve the science before changing clinical practice. *Radiology*.

[4] Sharma M, Pelz DM (2013). CT perfusion in acute stroke: added value or waste of time?. *Stroke*.

[5] Wintermark M, Zhu G, Patrie JT, Michel P (2013). Response to letter regarding article, ‘CT perfusion in acute stroke: added value or waste of time?’. *Stroke*.

[6] Huang AP-H, Tsai J-C, Kuo L-T (2014). Clinical application of perfusion computed tomography in neurosurgery. *Journal of Neurosurgery*.

[7] Kan P, Snyder KV, Binning MJ, Siddiqui AH, Hopkins LN, Levy EI (2010). Computed tomography (CT) perfusion in the treatment of acute stroke.. *World Neurosurgery*.

[8] Latchaw RE, Alberts MJ, Lev MH (2009). Recommendations for imaging of acute ischemic stroke: a scientific statement from the american heart association. *Stroke*.

[9] Snyder KV, Mokin M, Bates VE (2014). Neurologic applications of whole-brain volumetric multidetector computed tomography. *Neurologic Clinics*.

[10] Klingebiel R, Siebert E, Diekmann S (2009). 4-D imaging in cerebrovascular disorders by using 320-Slice CT. Feasibility and preliminary clinical experience. *Academic Radiology*.

[11] Orrison WW, Snyder KV, Hopkins LN (2011). Whole-brain dynamic CT angiography and perfusion imaging. *Clinical Radiology*.

[12] Salomon EJ, Barfett J, Willems PWA, Geibprasert S, Bacigaluppi S, Krings T (2009). Dynamic CT angiography and CT perfusion employing a 320-detector row CT: protocol and current clinical applications. *Clinical Neuroradiology*.

[13] Menon BK, O'Brien B, Bivard A (2013). Assessment of leptomeningeal collaterals using dynamic CT angiography in patients with acute ischemic stroke. *Journal of Cerebral Blood Flow and Metabolism*.

[14] Siebert E, Diekmann S, Masuhr F (2012). Measurement of cerebral circulation times using dynamic whole-brain CT-angiography: feasibility and initial experience. *Neurological Sciences*.

[15] Brouwer PA, Bosman T, Van Walderveen MAA, Krings T, Leroux AA, Willems PWA (2010). Dynamic 320-section CT angiography in cranial arteriovenous shunting lesions. *American Journal of Neuroradiology*.

[16] Siebert E, Bohner G, Dewey M (2009). 320-Slice CT neuroimaging: initial clinical experience and image quality evaluation. *British Journal of Radiology*.

[17] Gratama van Andel HAF, Venema HW, Majoie CB, Den Heeten GJ, Grimbergen CA, Streekstra GJ (2009). Intracranial CT angiography obtained from a cerebral CT perfusion examination. *Medical Physics*.

[18] Mendrik A, Vonken E, Van Ginneken B (2010). Automatic segmentation of intracranial arteries and veins in four-dimensional cerebral CT perfusion scans. *Medical Physics*.

[19] Smit EJ, Vonken E, van der Schaaf IC (2012). Timing-invariant reconstruction for deriving high-quality CT angiographic data from cerebral CT perfusion data. *Radiology*.

[20] Mendrik AM, Vonken EPA, De Kort GAP (2012). Improved arterial visualization in cerebral CT perfusion-derived arteriograms compared with standard CT angiography: a visual assessment study. *The American Journal of Neuroradiology*.

